# Health Risk Assessment Attributed to Consumption of Fish Contaminated with Mercury in the Rio Branco Basin, Roraima, Amazon, Brazil

**DOI:** 10.3390/toxics10090516

**Published:** 2022-08-31

**Authors:** Ana Claudia Santiago de Vasconcellos, Sylvio Romério Briglia Ferreira, Ciro Campos de Sousa, Marcos Wesley de Oliveira, Marcelo de Oliveira Lima, Paulo Cesar Basta

**Affiliations:** 1 Laboratory of Professional Education on Health Surveillance, Joaquim Venâncio Polytechnic School of Health, Oswaldo Cruz Foundation, Rio de Janeiro 21040-900, RJ, Brazil; 2 Postgraduate Program in Natural Resources (Pronat), Federal University of Roraima, Campus Paricarana, Boa Vista 69310-000, RR, Brazil; 3 Socioambiental Institute, São Paulo 01047-912, SP, Brazil; 4 Environmental Section, Evandro Chagas Institute, Secretariat of Science, Technology and Strategic Products, Ministry of Health of Brazil, Belém 70723-040, PA, Brazil; 5 Department of Endemic Diseases Samuel Pessoa, National School of Public Health Sergio Arouca, Oswaldo Cruz Foundation, Rio de Janeiro 21041-210, RJ, Brazil

**Keywords:** Amazon, fish consumption, gold mining, health risk assessment, methylmercury, Roraima

## Abstract

The aim of this study was to assess the health risk attributable to the consumption of mercury-contaminated fish for the urban and non-urban populations living in the Roraima state, Amazon, Brazil. Seventy-five fish specimens distributed across twenty different species, comprising four trophic levels (i.e., herbivore, omnivore, detritivore, and carnivore), were collected at four locations in the Branco River Basin. The fish samples were sent to the Toxicology Laboratory at Evandro Chagas Institute to determine the total-Hg levels by using the cold vapor atomic system (CVAAS). The total-Hg levels ranged from 0 to 3.159 µg/g. The average concentration in non-carnivorous species (*n* = 32) was 0.116 µg/g, and among carnivorous fish (*n* = 43), it was 0.869 µg/g. The weighted average of contamination levels for all samples was 0.545 µg/g. The health risk assessment was conducted according to the methodology proposed by the World Health Organization and different scenarios of human exposure were considered, based on three levels of fish consumption (low: 50 g/day; moderate: 100 g/day and high: 200 g/day). Women of childbearing age ingest 5 to 21 times more mercury than the dose considered safe by the U.S. EPA and intake a dose from 2 to 9 times higher than the safe dose proposed by FAO/WHO. Children under 5 years of age ingest from 18 to 75 times the dose proposed by the U.S. EPA and from 8 to 32 more mercury than the limit proposed by FAO/WHO. In summary, regardless of the level of fish consumption, type of residency (urban or non-urban), and the subset of the population analyzed, anyone who consumes fish from the locations sampled is at high risk attributable to mercury ingestion, with the only exception of adult men, who consume an average of 50 g of fish per day.

## 1. Introduction

Fish is a type of food with high nutritional value, due to its high protein content. Many fish species are rich in minerals, vitamins and polyunsaturated fatty acids that contribute to maintaining good health, preventing cardiovascular and neurological diseases [1,2]. Despite overwhelming evidence of the benefits associated with fish consumption, the growing contamination of Amazon aquatic systems by mercury used in gold mining (also called *garimpo* in Brazil) has led to discussions about the advantages and disadvantages of a diet rich in this kind of animal protein [3].

This economic activity is spread across the whole Amazon region, including on lands that belong to the federal government, such as conservation units, environmental protection areas and indigenous lands [4]. The fish contamination process occurs because a portion of the metallic mercury (Hg^0^) used by the miners during the process of gold extraction is converted into methylmercury (MeHg) by the action of microorganisms that live in the river sediment [5,6]. Over time, the methylmercury formed in the riverbeds is incorporated into all the organisms that comprise the aquatic biota, resulting in a phenomenon known as bioaccumulation [7]. Moreover, the organisms that sit at the top of the trophic chain, such as carnivorous fish, alligators, and aquatic mammals, accumulate higher methylmercury quantities in their tissues, due to the proven biomagnification capacity of this contaminant [8,9,10]. In this manner, the consumption of contaminated aquatic organisms is the main mercury exposure route for traditional Amazon populations [11,12].

Many studies conducted in the Amazon basin and elsewhere [7,13,14,15,16,17,18,19,20,21,22,23,24,25] have revealed that a large part of the fish captured in this region present methylmercury levels above the limits established by FAO/WHO [26] (in other words, 0.5 µg/g for non-carnivorous and 1.0 µg/g for carnivorous fish). Consequently, a large portion of the population living in the Amazon ingests methylmercury in amounts many times greater than the limits considered safe by FAO/WHO [27] and the U.S. EPA [28], for example, 0.23 µg/kg bw/day and 0.1 µg/kg bw/day, respectively. Recent review articles illustrate the magnitude of the problem by revealing the methylmercury levels detected in hair samples of individuals from different locations in the Brazilian Amazon [29,30].

The consumption of fish contaminated by mercury can cause different effects on human health in the short and long term. In Amazon children exposed during the first few months of the prenatal period, loss of cognitive ability, psychomotor alterations and problems in mental development have been observed [31,32,33]. On the other hand, in chronically exposed adults, symptoms such as depression, aggressiveness, insomnia, problems in motor coordination and visual capacity are commonly described [34,35].

In addition to the advancement of gold mining in the Amazon territory, the building of dams for hydroelectric plants also affects the mercury cycle in this environment. Dams facilitate the mercury methylation process (i.e., formation of methylmercury) and, additionally, inhibit the migration of fish, facilitating bioaccumulation and biomagnification of the methylmercury produced [36,37,38,39,40].

It is important to keep in mind that, for social and economic reasons, the traditional peoples of the Amazon, such as indigenous peoples and riverside dwellers, are some of the largest consumers of fish on the planet. There are studies that point to an average fish consumption by riverside-dwelling communities of more than 300 g per day and that annual average consumption can exceed 100 kg per inhabitant [41]. A direct consequence of the high fish consumption is the high mercury contamination prevalence, as has been previously reported in several studies [4,29,30,42,43]. On the other hand, there are few studies that focus on the exposure of populations that live in the urban areas of the Amazon [44,45,46].

Considering the progressive encroachment of the Amazon by illegal gold miners and the serious health damage that mercury can cause, the main objective of this work was to assess the risk attributable to the consumption of mercury-contaminated fish from the Branco River Basin for urban and non-urban populations living in the Roraima state, north of the Brazilian Amazon.

Therefore, the leading novelty of this approach is the investigation of the mercury levels in freshwater fish collected in the Rio Branco Basin and commercialized in the markets of the main cities in the Roraima state, as well as assessing the risk of fish consumption in the health of the traditional people from the Amazon.

## 2. Materials and Methods

### 2.1. Study Area

The Branco River is the largest river of the Roraima state, and the largest tributary of the Negro River. This Basin covers 83% of Roraima, with a drainage area that covers 187,540 km^2^, a part of which (12,310 km²) is formed in Guyana territory [47]. The Branco River supplies the capital and most of the state’s population. It sustains agriculture and livestock raising and serves as a source of income for the 4000 people who make their living from fishing. Moreover, it is a tourist spot for the enjoyment of all the population.

Although gold exploration in the state of Roraima began decades ago, causing contamination of fish and, consequently, the mercury exposure of the populations living in the region [10,48,49,50], the last few years have been marked by an overwhelming growth of this activity. The significant environmental devastation of the Amazon rainforest has affected several local aquatic systems, including the Mucajaí and Uraricoera rivers that form part of the Rio Branco Basin [51,52].

### 2.2. Health Risk Assessment

A health risk assessment attributable to the consumption of mercury-contaminated fish was conducted in accordance with the methodology proposed by the World Health Organization (WHO, 2008) [53]. This methodology is divided into the following four main steps:

(i) The first step is descriptive and consists, basically, of the characterization of the population studied (i.e., the definition of the population subgroups and their respective average body weights) to determine the mercury levels in fish consumed and estimate the quantity of fish ingested daily.
(ii) The second step concerns the calculations used to estimate the average mercury quantity ingested daily by the studied population subgroups. In other words, in this step, we calculated the daily dose of mercury ingested.
(iii) The third step consists of calculating the risk ratio. To do so, the daily dose of mercury ingested is divided by the recommended safe dose (or reference dose) of a health agency (e.g., FAO/WHO [27], U.S. EPA [28]).
(iv) The final step refers to the creation of distinct exposure scenarios for a counterfactual analysis. The counterfactual analysis is designed to investigate the impact of hypothetical situations that could potentially reflect real exposures. In the context of this approach, the counterfactual analysis evaluates the impact of different levels of consumption of mercury-contaminated fish on the selected population subgroups’ health.

#### 2.2.1. First Step in the Risk Assessment

##### Collection and Storage of Fish Samples

**Figure 1:**
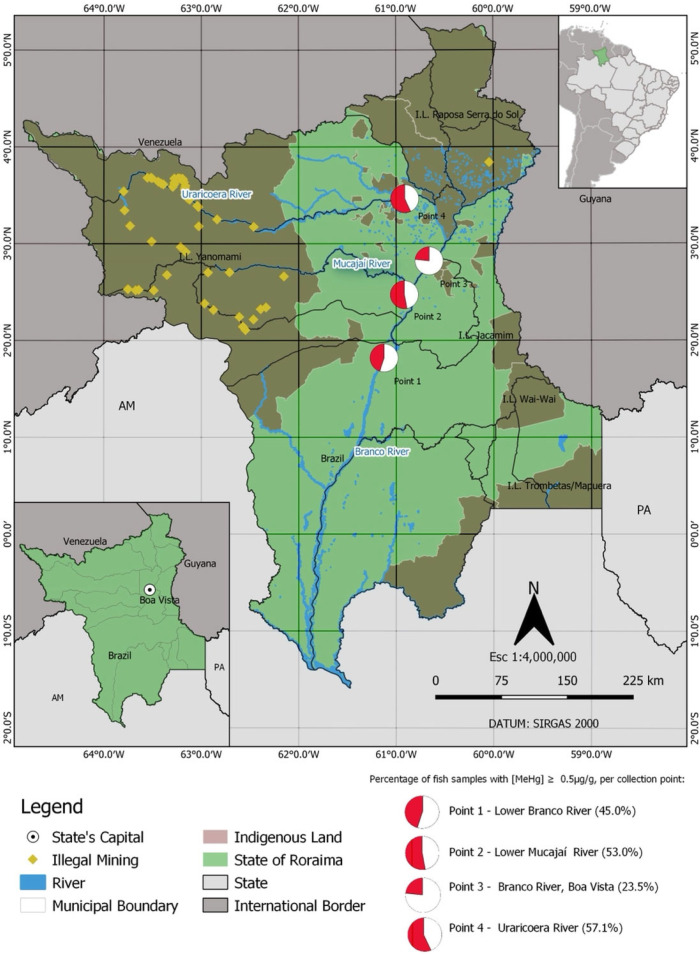
Map of the state of Roraima, highlighting the four fish collection points. Source: Cartographic Bases (1:250,000) of the Brazilian Institule of Geography and Statistics (IBGE) and Amazonian Network of Geo-referencedSocio-environmental Information(RAISG). Available online: https://www.amazoniasociambiental.org/pt.br/; https://www.ibge.gov.br/geociencias/cartas-e-mapas/based-cartograficas-continuas/15759-brasil.html?=downloads, accessed on 15 February 2022.

Fish were collected from the following four locations in the Branco River Basin (Figure 1): Collection Point 1—Lower Branco River (01°49′08″ N/61°07′29″ W); Collection Point 2—Lower Mucajaí River, right bank tributary of the Branco River (02°28′19″ N/60° 54′ 57″ W), Collection Point 3—Branco River in the capital Boa Vista (2°49′17″ N/60°39′42″ W), and Collection Point 4—Uraricoera River, in the municipality of Amajari (03°27′48″ N/60°54′38″ W). These collection sites represent the main fishing landing points in the region and supply supermarkets and street markets in the municipalities. In addition, they have different degrees of influence from mining activity. The sampling points located on the Uraricoera and Mucajaí rivers are in more gold mining-impacted areas than the points located on the Branco River and, consequently, have greater potential for mercury contamination [51,52].

The fish were acquired directly from fishermen at landing points, between 27 February and 6 March 2021. After acquisition, the fish were immediately packaged in a cooler with ice and shipped to Boa Vista. Each sample was photographed, and the information on collection noted on field record cards with the following data: common name, provisional scientific name, date, fishing location, collection location, geographic coordinates, tools employed, standardized weight (g), and length (cm).

The taxonomic identification of each specimen collected was confirmed to the maximum degree possible using a specialized bibliography, dichotomous keys, and consultation with specialists. Afterward, around 6 g of muscle tissue was extracted from the dorsal part of each specimen, which was divided into 3 portions of roughly 2 g. The samples were placed cryotubes, packaged in appropriate boxes and frozen in a freezer at approximately −17 °C. Duplicates and triplicates were also identified on the field record card, packaged in insulated boxes with ice, and shipped, by air, to Evandro Chagas Institute (IEC) [54].

##### Mercury Analysis

The total mercury (THg) determination in fish samples was carried out at the Toxicology Laboratory at the Evandro Chagas Institute (IEC), following the methodology proposed by Akagi et al. [55]. For each sample, 0.3 to 0.5 g of muscle tissue was weighed (wet weight) in a 50 mL Pyrex^®^ volumetric flask (Corelle Brands, Charleroi, Belgium). Then, 1 mL of deionized water, 2 mL of HNO_3_ and HClO_4_ (1:1), and 5 mL of H_2_SO_4_ were added for digestion. The vials were exposed to a hot plate (200 to 230 °C) for 30 min. After cooling to room temperature, the flasks were measured with deionized water and the digested samples were homogenized. The THg determination was carried out using the cold vapor atomic absorption system (CVAAS), using semi-automatic mercury analyzer equipment Analyzer Model Hg-201 (Sanso Seisakusho Co. Ltd., Tokyo, Japan). To guarantee the quality assurance (QA)/quality control (QC), for the mercury analysis of the fish samples, we used the following parameters: (i) reference materials of dogfish liver-certified reference material for trace metals (DOLT-4) (% of recovery: 92.24 ± 7.73; 70.92 to 100) and fish protein-certified reference material for trace metals (DORM-3) (% of recovery: 96.22 ± 4.69; 87.16 to 100) from the National Research Council of Canada; (ii) a method blank; (iii) a 6-point calibration curve; and (iv) the relative standard deviation (RSD) of 8.32%. The detection and quantification limits (LOD/LOQ) obtained were 0.0083 ng/mg and 0.027 ng/mg, respectively.

For this study, we assume that the entire mercury content detected in the fish samples is in the form of methylmercury, since, as previously described, more than 90% of the mercury species in fish muscle samples are present in this chemical form [56,57].

##### Average Concentration of Methylmercury in Fish

Assuming as a working hypothesis that the diet of urban and non-urban populations of the state of Roraima is composed of different fish, without any preference for a specific species, and that fish from different capture locations are consumed (i.e., from different rivers that form the Branco River Basin), the weighted average of methylmercury detected in carnivorous and non-carnivorous fish (i.e., herbivorous, omnivorous, detritivorous) was used as the average concentration.

##### Definition and Characterization of Population Subgroups

Considering that the potentially deleterious effect of methylmercury on the human body varies according to the age and sex of the individuals, the following population subgroups were selected for analysis: (i) women of childbearing-age (10 to 49 years); (ii) adult men (aged ≥18 years); (iii) children aged 5 to 12 years; and (iv) children aged 2 to 4 years.

In addition, in view of the possibility that urban and non-urban populations of the state of Roraima could present different anthropometric profiles and since different profiles can influence the interpretation of results of the risk assessment, the study opted to analyze these groups separately.

The study used weight data for urban and non-urban populations that reside in the northern region of Brazil, obtained by consulting the Household Budget Survey [58], available from IBGE’s Automatic Recovery System.

For the urban population subgroups, the following average weights were used: 51.27 kg for women of childbearing-age; 68.59 kg for adult men; 28.82 kg for children aged 5 to 12 years and 14.85 kg for children aged 2 to 4 years. For the non-urban population subgroups, the following average weights were used: 50.64 kg for women of childbearing-age; 65.17 kg for adult men; 27.03 kg for children aged 5 to 12 years and 14.48 kg for children aged 2 to 4 years.

##### Average Amount of Fish Ingested

Considering that there is no official information on the average consumption of fish by the urban and non-urban populations of the state of Roraima, the following three hypothetical patterns of fish consumption were established: (i) low consumption—50 g per day; (ii) moderate consumption—100 g per day; and (iii) high consumption—200 g per day. These diet patterns were used to create exposure scenarios for the counterfactual analysis.

#### 2.2.2. Second Step in the Risk Assessment

##### Estimated Methylmercury Ingestion (Ingestion Dose)

To estimate the daily ingestion of methylmercury, the following two assumptions were made: (i) 100% of the mercury detected in the fish samples is in the chemical form of methylmercury (MeHg); and (ii) 100% of the methylmercury present in the fish muscle is absorbed by the human gastrointestinal tract after consumption. The amount of mercury ingested was estimated using the following formula:

(1) $$MI=\frac{FI\times MC}{BW}$$

where

*MI* is the average amount of methylmercury ingested per kilogram of body weight per day (µg/kg bw/day);

*FI* is the amount of fish ingested per day (g/day);

*MC* is the average mercury concentration in the fish ingested (µg/g)**;**

*BW* is body weight (kg)**.**

#### 2.2.3. Third Step in the Risk Assessment

##### Risk Ratio Calculation

The calculation of the risk ratio was based on the ratio of the estimated mercury ingested (or estimated ingestion dose) and the reference doses proposed by the FAO/WHO [27] or U.S. EPA [28].

When the risk ratio is below 1, the methylmercury ingestion dose is below the reference doses; consequently, the risk of illness is low. On the other hand, when the risk ratio is above 1, the dose of methylmercury ingested exceeds the reference doses and, for this reason, the risk of illness due to exposure to mercury must be considered.

(2) $$RR=\frac{MI}{RD}$$

where

*RR* is the risk ratio (RR < 1.0 = remote risk of illness/RR ≥ 1.0 = real risk of illness);

*MI* is the estimated methylmercury ingestion dose (µg Hg/kg bw/day);

*RD* is the reference dose (0.1 µg Hg/kg fish/day/0.23 µg Hg/kg bw/day/0.45 µg Hg/kg bw/day).

#### 2.2.4. Fourth Step in the Risk Assessment

##### Creation of Exposure Scenarios and Counterfactual Analysis

For the counterfactual analysis, the following three methylmercury exposure scenarios were created, considering the different patterns of fish consumption determined hypothetically: “Scenario 1—low consumption of fish: 50 g fish/day”; “Scenario 2—moderate consumption of fish: 100 g/day” and “Scenario 3—high consumption of fish: 200 g/day”.

### 2.3. Maximum Safe Consumption of Fish (MSC)

The calculations to define the maximum safe fish consumption were carried out considering the reference doses established by the FAO/WHO [27] and U.S. EPA [28], according to the formula presented below.

(3) $$MSC=\frac{\left(RD\times BW\right)}{MC}$$

where

*MSC* is the maximum amount of fish (grams per day) that can be safely consumed (considering the different reference doses for methylmercury);

*RD* is the dose reference used as the safe limit for the ingestion of methylmercury (i.e., dose recommended by the U.S. EPA: 0.1 µg Hg/kg bw/day; dose recommended by FAO/WHO: 0,23 µg Hg/kg bw/day for childbearing-age females and children; 0.45 µg Hg/kg bw/day for adults in general);

*BW* is average body weight (kg);

*MC* is the average of mercury concentration in the fish (µg/g).

## 3. Results

### 3.1. Fish Collection and Contamination with Methylmercury

In total, 75 fish specimens were collected, comprising of 20 species and 4 trophic levels (i.e., herbivorous, omnivorous, detritivorous, and carnivorous). At the collection point on the Branco River, in the city of Boa Vista (Collection Point 3), 17 fish were collected with an average mercury concentration of 0.31 µg/g (SD 0.29) and median concentration of 0.17 µg/g. At the collection point situated in the region of the Lower Branco River (Collection Point 1), 20 fish were collected with an average mercury concentration of 0.72 µg/g (SD 0.71) and median concentration of 0.56 µg/g. At the Lower Mucajaí River (collection point 2), 17 fish were collected with an average mercury concentration of 0.43 µg/g (SD 0.42) and median concentration of 0.45 µg/g. Finally, at the Uraricoera River (Collection Point 4), 21 fish were collected with an average mercury concentration of 0.68 µg/g (SD 0.82) and median concentration of 0.42 µg/g (Table 1).

Considering only the median values for mercury concentration, the fish contamination level by collection point is (in ascending order) as follows: Collection Point 3 (Branco River—Boa Vista) < Collection Point 4 (Uraricoera River) < Collection Point 2 (Mucajaí River) < Collection Point 1 (Lower Branco River). Analysis of mercury contamination prevalence (MeHg ≥ 0.5 µg/g) by collection point reveals that at Collection Point 3 (Branco River, Boa Vista), 23.5% of the fish collected present mercury concentrations above the limit; at Collection Point 1 (Lower Branco River), 45.0%; at Collection Point 2 (Mucajaí River), 53.0% and at Collection Point 4 (Uraricoera River), 57.1%.

The mercury concentration average in non-carnivorous species (*n* = 32) was 0.116 µg/g (SD 0.126) and the average concentration for carnivorous fish (*n* = 43) was 0.869 µg/g (SD 0.655). The weighted average of contamination levels in these two groups was, therefore, 0.545 µg/g.

**Figure 2:**
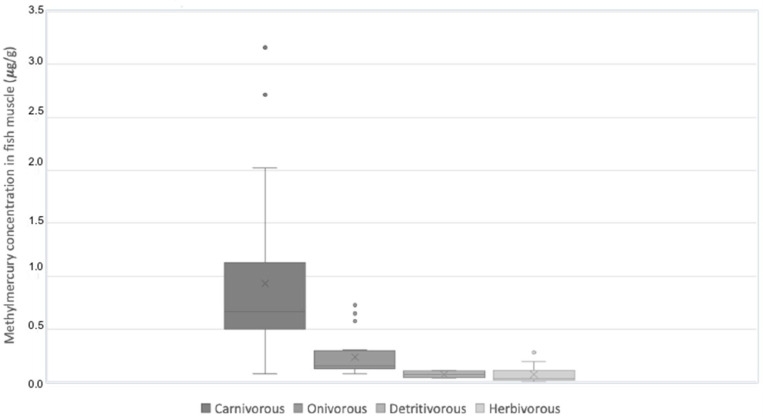
Methylmercury concentration in fish samples according to trophic level.

The capacity of methylmercury for biomagnification throughout the trophic chain was confirmed and is shown in Figure 2. The medians of methylmercury concentrations detected in the different trophic levels were, in descending order, as follows: 0.66 µg/g in carnivorous fish (average: 0.93; SD: 0.68); 0.15 µg/g in omnivorous fish (average: 0.23; SD: 0.20); 0.068 µg/g in detritivorous fish (average: 0.07; SD: 0.03) and 0.03 µg/g in herbivorous fish (average: 0.07; SD: 0.08).

### 3.2. Health Risk Assessment

Table 2 presents the methylmercury doses ingested by different population subgroups, according to the three fish consumption patterns proposed (i.e., low, moderate, and high). Only the subgroup compounded of “adult men”, with low fish consumption, was able to ingest a methylmercury dose below the limit established by FAO/WHO (i.e., 0.45 µg/kg bw/day). However, when we consider the U.S. EPA reference dose (i.e., 0.1 µg/kg bw/day) all populations, without exception, ingest methylmercury in amounts above the limit considered safe.

Table 3 presents the risk ratio attributable to the consumption of methylmercury contaminated fish in different exposure scenarios. The estimates reveal that methylmercury ingestion exceeds the limits recommended by the U.S. EPA [28] and FAO/WHO [27] in practically all the created scenarios. The exceptions were the two scenarios created for the adult men’s subgroup with low fish consumption, in which the reference dose used for the calculation was that established by FAO/WHO. In this case, the methylmercury doses ingested based on fish consumption of 50 g are below the limit of 0.45 µg/kg bw/day, as recommended by this health agency.

### 3.3. Maximum Safe Fish Consumption

Table 4 indicates the safe amount of fish (g/day) that can be consumed by different urban and non-urban population subgroups, according to the dose limit proposed by the U.S. EPA [28]. In this case, women of childbearing-age and residents of urban and non-urban areas can consume amounts above 50 g per day of the following species of fish: Curimatã, Jandiá, Jaraqui and Pacú. The safe amounts for ingestion of fish range from 55 g to 170 g per day, depending on the species, while adult males can consume Aracú, Curimatã, Jandiá, Jaraqui, Matrinxã and Pacú, in amounts that range from 50 to 228 g. Children aged 5 to 12 years of age can consume Jaraqui and Pacú in maximum amounts that range from 69 to 96 g per day. Finally, it is not recommended that children aged 2 to 4 consume the species of fish collected in this study in amounts above 50 g per day.

The safe amount of fish for consumption, according to the reference dose from FAO/WHO [27], can be found in Table 5. Women of childbearing-age can consume Aracú, Curimatã, Jandiá, Jaraqui, Matrinxã and Pacú, in amounts that range from 85 to 393 g per day, depending on the species. Adult men can consume Aracú, Curimatã, Jandiá, Jaraqui, Liro, Mandi, Matrinxã, Pacú and Piranha-preta in amounts that range from 66 to 1028 g per day. Children aged 5 to 12 can consume Aracú, Curimatã, Jandiá, Jaraqui, Matrinxã and Pacú in daily amounts that range from 50 to 220 g while children aged 2 to 4 can consume Jaraqui and Pacú, in amounts that range from 85 to 113 g per day.

## 4. Discussion

As far as we know, this is the first study designed to assess the health risk attributable to the consumption of mercury-contaminated freshwater fish from the Branco River Basin, in the state of Roraima.

In spite of the many benefits associated with the regular inclusion of fish in one’s diet, such as lower levels of cholesterol in the blood, lower risk of heart attack, and improved cognitive development, the contamination of fish with methylmercury represents a real threat that cannot be ignored by health authorities [59,60,61].

This study revealed that the fish collected in the Branco River Basin presented levels of mercury that ranged from 0 to 3.16 µg/g. Of the four collection points selected to obtain the fish, three presented a high prevalence of contamination. Practically half the fish collected from the Lower Branco River (45.0%), from the Mucajaí River (53.0%) and from the Uraricoera River (57.1%) presented concentrations of methylmercury equal to or above the limit established by FAO/WHO (i.e., 0.5 µg/g Hg) for the sale of fish. The high rates of contamination observed are probably the result of the numerous illegal gold mining operations established along with the Mucajaí and Uraricoera Rivers [51,52]. Reinforcing this hypothesis, the fish collected from the Uraricoera River presented the highest mercury levels (Collection Point 4). At that collection point, for each 10 fish collected, 6 presented mercury levels ≥ 0.5 µg/g. In turn, at the Collection Point 1, located on the Branco River in the capital of Boa Vista, the lowest levels of contamination (23.5%) were reported. In this case, for each 10 fish collected, approximately 2 presented mercury levels ≥0.5 µg/g. The lower levels of contamination may reflect the greater distance from the illegal gold mining operations across the Yanomami Indigenous Land.

In Brazil, the National Health Surveillance Agency (ANVISA) establishes the maximum limits for various chemical contaminants in foods. Regarding the presence of contaminants in fish, ANVISA Resolution n°. 42 establishes that the maximum limit for inorganic mercury in fish is 0.5 µg/g for non-predatory species and 1.0 µg/g for predatory species [62]. Considering that almost all mercury present in fish muscle is in the form of methylmercury (i.e., an organic form of mercury), we can say that a resolution that establishes limits for inorganic mercury species is not appropriate to guarantee safe information about fish consumption to the society. So, we suppose that the ANVISA resolution is just a misguided adaptation of the limits recommended by FAO/WHO for methylmercury in fish (i.e., 0.5 µg/g for non-carnivorous fish and 1.0 µg/g for carnivorous fish). It is important to note that the limits established by FAO/WHO, adopted in 1991 [26], are applicable only to fish commercialization and do not consider the health effects produced by the ingestion of methylmercury in fish. The calculations to determine these maximum limits were conducted based on data on the average levels of mercury in samples of fish from different trophic levels.

When we compare the results of the present study with the work conducted by Sing et al. [48] approximately 20 years ago, we observe that the progressive encroachment of gold mining activity in Roraima has contributed to a significant increase in mercury levels in fish. In 2003, Sing et al. [48] conducted a study in Yanomami communities that live along the banks of the Catrimani and Ajarani rivers, which revealed mercury levels in predatory fish samples that range from 0.23 µg/g to 1.08 µg/g. The maximum limit detected by the authors [48] was three times lower than that observed in the fish collected in the Branco River Basin in this study. The authors also included evidence about the contamination of indigenous people, since mercury levels in blood samples, ranging from - to 62.6 µg/L (which is equivalent to 12 µg/g in hair) were found among the participants.

In another study conducted with indigenous people of the Makuxi ethnic group, Sing et al. [49] found lower levels of contamination (ranging from 2.0 to 31.3 µg/L in blood samples). However, this lower level of contamination probably reflects the reduced consumption of fish by this ethnic group and does not necessarily mean it is a result of the lower environmental contamination by the mercury resulting from mining. In turn, the Yanomami mercury contamination has also been reported by Castro et al. [50] in the regions of Surucucu, Paapiu and Mucajaí, in the state of Roraima. The authors report that 40% of the participants presented hair mercury concentrations above 6.0 µg/g. Finally, in a more recent study, commissioned by the Hutukara Yanomami Association, Vega et al. [43] identified median concentrations of mercury in the hair of Yanomami who live in the regions of Waikás-Aracaça and Paapiu of 15.5 µg/g and 3.2 µg/g, respectively. The difference in the values is due to the fact that in Waikás-Aracaça, gold extraction operations are fully operational, whereas in Paapiu, the gold mining operations were decommissioned in the 1980s. Nevertheless, at the time of data collection, in 2014, approximately 7% of the participants presented mercury levels above 6,0 µg/g in the hair samples, revealing the enduring presence of methylmercury in the environment, particularly in the food chain.

The studies mentioned above show that gold mining activity has been present in the state of Roraima for decades, primarily on indigenous lands where it is illegal, resulting in the progressive contamination, not only of the environment and the fish of the region, but also of the human and non-human populations that use fish as a source of food protein in their diet.

The risk ratios estimated in this study indicate that in almost all scenarios proposed for the counterfactual analysis, there is no safe level of fish consumption for the populations that live in Roraima and consume fish from the Branco River Basin. This means that regardless of the fish consumption level (i.e., low, moderate, or high), whether the population is urban or not, and regardless of the population subgroup analyzed (i.e., childbearing-age woman, children, or adult men), everyone who consumes fish from the sample collection locations is at high risk from the ingestion of methylmercury. According to the U.S. EPA [28], women of childbearing-age, the population most vulnerable to the methylmercury action, ingest 5 to 21 times more mercury than the dose considered safe. Considering the limits established by FAO/WHO [27], the amount ingested is 2 to 9 times greater than the dose considered safe. For children under 5, the results are even more alarming. Considering the U.S. EPA reference dose [28], the ingestion of mercury is 18 to 75 times higher than the limit proposed, and according to FAO/WHO [27], the ingestion amount is 8 to 32 times higher.

Comparing the risk ratios estimated for the urban and non-urban populations, we can observe that both are equally at risk of illness, due to the ingestion of fish contaminated with mercury. Despite the urban population presenting slightly higher weighted averages than the averages of the non-urban population and, consequently, ingesting slightly lower doses of methylmercury daily, this difference is not significant for reducing the risk of illness. We can conclude based on the risk analysis that the entire population that eats fish from the Branco River Basin is at risk from the ingestion of methylmercury in doses above the parameters proposed by FAO/WHO [27] and U.S. EPA [28], with the only exception of adult men, who can consume an average of 50 g of fish per day. Similar results were obtained in risk assessment studies that involved traditional communities in the state of Amapá, located in the north of the Amazon [19], population groups affected by *garimpos* in Colombia [63], and Munduruku indigenous people living in the region of the middle Tapajós River, in the state of Pará [13].

The parameters proposed by these health agencies are based on data obtained from longitudinal studies conducted in the Faroe and Seychelles Islands, as well as New Zealand [64,65,66,67]. Despite the relevance and the consistency of these studies, the locations where the studies were conducted differ greatly from the Amazon environment and people who live there. The Amazon ecosystem presents characteristics that create a unique exposure scenario and includes not only the presence of natural mercury in the soil, but also mercury from anthropic action, marked by the construction of dams for hydroelectric plants, and by expanding deforestation, burn-offs and gold mining. All these factors alter the natural cycle of mercury in the environment and, consequently, increase the risk of exposure to the human populations that have inhabited the Amazon since ancient times [3,37,38,39].

The group most vulnerable to the toxic effects of methylmercury is compounded of pregnant women and their babies, since the damage inflicted by this contaminant on the central nervous system of the fetus is extensive, including motor and sensory deficits, in addition to cognitive losses, which in most cases are irreversible. Neurotoxicity in fetuses gained international attention after the Minamata tragedy in the 1950s and 1960s [68]. Years later, cohort studies conducted in the Faroe Islands and in New Zealand showed that even at low doses, the consumption of fish contaminated with methylmercury during pregnancy can cause important cognitive losses in children [64,67].

In agreement with the above-mentioned findings, Reuben et al. [31], in a study conducted in the Peruvian Amazon, revealed that children exposed to mercury as a result of goldmining from as early as the prenatal period present an important loss of cognitive capacity. In turn, Marques et al. [32,33] identified psychomotor alterations and mental development problems in children submitted to the same exposure scenario, in the state of Rondônia, in the Brazilian Amazon.

In adult individuals, the symptoms related to mercury exposure can be expressed in a different way. Recent studies have outlined the emotional changes, such as depression and aggressiveness, motor problems [34] and changes in the visual field [35]. In addition, some studies have shown an association between exposure to methylmercury and the development of neuro-degenerative diseases, such as Alzheimer’s and Parkinson’s [69,70]. Moreover, studies conducted with Munduruku indigenous people who live in the region of the Middle Tapajós showed neurological and psychological changes in adults and delays in the development of children associated with the consumption of mercury-contaminated fish [71,72,73].

In addition to damage to the central nervous system, consumption of methylmercury-contaminated fish can also cause changes in the cardiovascular system. Hu et al. [74], after conducting a meta-analysis on this topic, suggested that hair mercury concentrations between 2.0 and 3.0 µg/g can be considered as borderline concentrations for the risk of developing hypertension. In Brazil, Fillion et al. [75], while studying riverside-dwelling communities in the Amazon, found that people with hair mercury levels above 10 µg/g had almost a 3 times greater risk (RC: 2.91; CI95% 1.26–7.28) of developing high levels of systolic blood pressure compared to people with lower levels of mercury exposure. Likewise, Salonen et al. [76], in a longitudinal study with Finnish men, concluded that hair mercury levels above 2.0 µg/g represented a 69% higher risk of suffering an acute heart attack.

To illustrate the size of the economic, social, environmental, and health impact on the populations affected by chronic mercury exposure from the illegal activities of gold mining in the Amazon, Bakker et al. [77] developed a methodology entitled “mercury impact calculator”. This calculator was developed to be used by the federal prosecutor’s office as a tool to estimate the size of the economic, social, environmental and health damage on the populations affected, with a view to levying fines on the perpetrators. To develop this tool, the authors considered the consequences of contamination, based on Hg exposure doses from hair samples, and the development of the following selected health outcomes: neurodevelopmental delays, acute heart attack and systemic arterial hypertension. Using the concept of disability-adjusted life years (DALY), the authors report that economic losses could reach USD 400,000 for each kilogram of gold extracted illegally. Putting this tool in practice, the authors used the expansion of gold mining on the Yanomami Indigenous Land as a case study in 2020 and found that the losses resulting from contamination by mercury, from the illegal mining of gold, totaled 69 million dollars, in just one year. The authors concluded that if there was regular and effective environmental surveillance and if national legislation was enforced, these resources could be invested on behalf of the local population, through sustainable development projects and infrastructure works, with social participation and prior consultation, to meet the real needs of the communities.

Finally, despite the illustrative findings of this investigation, it is important to mention some limitations. The data analyzed here are from 75 fish specimens, collected from only 4 collection points, in the Branco River Basin. Despite the collective effort of the authors to acquire the fish species most frequently consumed by the local population, the sample number is small and cannot be considered representative of all the populations of fish available throughout the Branco River Basin. Another important point is that our collection occurred at the end of the dry season in Roraima, and there was no way of collecting fish during the rainy season. Despite the sample limitations, the four collection points included in this study represent the produce unloaded from fishing boats in strategic locations of the state, both with regard to the purchase and sale of fish for consumption by the local population, and as a proxy to assess the extent of contamination by mercury, from the regions most impacted by illegal gold mining in the Yanomami Indigenous Land, in other words, the Uraricoera and Mucajaí rivers beds that serve several municipalities, and therefore reach the urban centers of the state.

Another important limitation of this study concerns the calculations for risk estimation. To carry out the risk assessment attributable to the consumption of mercury-contaminated fish, we assume that 100% of the mercury present in the muscle of the fish collected is in the form of methylmercury, and 100% of this mercury is absorbed by the human gastrointestinal tract. These two assumptions overestimate the calculated risk, basically, for two main reasons, which are as follows: (i) recent studies indicate that the THg/MeHg ratio varies considerably among fish species [78,79] and (ii) the absorption of mercury by the human body can vary for different reasons and among them is the method of cooking the fish [80]. Despite the risk presented in this study being overestimated, the doses of methylmercury intake are at least twice the limit proposed by the U.S. EPA and FAO/WHO in most of the scenarios constructed. This means that, despite the limitations exposed, the results of this study clearly indicate that the fish collected in the Rio Branco Basin have levels of mercury that put the local population at high risk.

The three counterfactual scenarios used to estimate risk are also hypothetical and may not represent the true consumption of families. On one hand, it is possible that urban families consume less than 50 g per day and, as such, health risks are lower. On the other hand, it is possible that for some rural populations, consumption is higher than 200 g per day, seriously increasing the health risks. For this reason, our data must be interpreted with caution. Nevertheless, if this methodology for analyzing health risks attributable to the consumption of contaminated fish is expanded to include other areas of Roraima, the risks are likely to be greater, and the situation is even more serious than reported here.

## 5. Conclusions

As long as the illegal extraction of gold in the Amazon and its legacy of environmental destruction persists, one viable strategy to minimize human exposure to mercury is to prioritize the consumption of species with low mercury concentrations. Fish such as aracú, curimatã, jaraqui, matrinxã and pacú have low mercury concentrations (because they are non-carnivorous species) and, therefore, can be consumed in amounts greater than 50 g per day, even by women of childbearing age and children. On the other hand, carnivorous fish, such as surubim, filhote/piraíba, pescada and tucunaré, widely consumed by the population of Roraima, should be avoided by women of childbearing age and children. Other population groups, such as adult men and women over 49 years of age, should consume these species in a restricted way, not exceeding one meal a month. It is important to alert the population about the consumption of fish from fish farming, which often can cause health risks. As some authors have reported [81,82] many times, the site used for fish farming is a site previously used for mining and has significant levels of mercury contamination.

In conclusion, considering the findings reported here and aiming to obtain even more consistent results, we suggest that future studies on the risk health assessment should consider the variations in methylmercury concentrations, according to the investigated fish species. Even though the detection of methylmercury concentration in fish muscle provides more reliable information on the dose of methylmercury ingested, we know that in the Amazon region, few laboratories have appropriate infrastructure for this type of analysis. Furthermore, we suggest that future studies consider the influence of the fish cooking method on the absorption of methylmercury from the gastrointestinal tract in order to enhance the quality of the risk estimation calculations.

## Figures and Tables

**Table 1:** Characterization of the fish collected in the Branco River Basin, Roraima, Brazil.

Common Name (*n*)	Average (Hg) μg/g	Standard Deviation	Min–Max Hg	Average Weight (g)	Min–Max Weight (g)	Average Total Length (cm)	Min–Max Total Length	Trophic Level
Aracu (05)	0.14	0.11	0.01–0.28	336	153–500	29.7	25–32.8	Herbivorous
Barba Chata (03)	2.07	0.57	1.60–2.72	868	555–1120	47.3	41.5–51	Carnivorous
Cará-açú (05)	0.40	0.20	0.17–0.65	694	511–963	36.3	27–50	Omnivorous
Coroataí (04)	2.01	0.9	0.96–3.16	1378	422–4085	51.8	39–84	Carnivorous
Curimatã (03)	0.09	0.02	0.07–0.11	358	265–405	28.1	26–29.7	Detritivorous
Dourada (02)	0.66	0.04	0.63–0.69	1280	955–1605	80.5	61–100	Carnivorous
Filhote/Piraíba (03)	1.17	0.31	0.87–1.49	18,348	3045–32,000	99	68–115	Carnivorous
Jandiá (02)	0.09	0.03	0.07–0.11	1111	1090–1132	49.8	49.5–50	Carnivorous
Jaraqui (02)	0.04	0.01	0.04–0.04	380	380	29	28.5–29.5	Detritivorous
Liro (01)	0.43	N.A.	N.A.	354	N.A.	38	N.A.	Carnivorous
Mandii (02)	0.44	0.40	0.15–0.72	67	41–92	21	18–24	Omnivorous
Mandubé (01)	0.62	N.A.	N.A.	580	N.A.	39	N.A.	Carnivorous
Matrinxã (12)	0.13	0.03	0.07–0.2	358	232–490	27.9	25–30	Omnivorous
Pacú (08)	0.03	0.02	0.00–0.07	334	131–670	23.4	19–30.1	Herbivorous
Pescada (07)	0.61	0.22	0.47–1.08	564	395–680	36.4	30.5–39	Carnivorous
Piracatinga (01)	1.47	N.A.	N.A.	770	N.A.	45.6	N.A.	Carnivorous
Pirandirá (01)	1.06	N.A.	N.A.	2025	N.A.	56.6	N.A.	Carnivorous
Piranha-preta (02)	0.40	0.03	0.38–0.42	227	176–278	21.8	20.5–23	Carnivorous
Surubim (06)	0.65	0.18	0.43–0.95	1163	885–1905	54.2	50–63.3	Carnivorous
Tucunaré (05)	0.71	0.26	0.49–1.12	950	615–1605	37.4	34.3–52.5	Carnivorous

**Table 2:** Estimates of mercury doses ingested according to different fish consumption levels and population subsets.

				Dose of Hg Ingested (µg/g)	
Average (Hg) in Fish (µg/g)	Level of Consumption	Mass of Hg Ingested (µg)	Population Subset	Urban Population	Non-Urban Population
0.545 µg/g	Low consumption (50 g/day)	27.26	Women of childbearing age	0.53	0.54
			Adult Men	0.40	0.42
			Children aged 5 to 12	0.95	1.01
			Children aged 2 to 4	1.84	1.88
	Moderate consumption (100 g/day)	54.52	Women of childbearing age	1.06	1.08
			Adult Men	0.79	0.84
			Children aged 5 to 12	1.89	2.02
			Children aged 2 to 4	3.67	3.77
	High consumption (200 g/day)	109.04	Women of childbearing age	2.13	2.15
			Adult Men	1.59	1.67
			Children aged 5 to 12	3.78	4.03
			Children aged 2 to 4	7.34	7.53

**Table 3:** Risk ratio attributable to the consumption of fish contaminated by mercury according to the reference doses proposed by the U.S. EPA and FAO/WHO.

	Risk Ratio U.S. EPA						Risk Ratio FAO/WHO					
	Consumption of Urban Population			Consumption of Non-Urban Population			Consumption of Urban Population			Consumption of Non-Urban Population		
	Low	Moderate	High	Low	Moderate	High	Low	Moderate	High	Low	Moderate	High
Women of childbearing age	5.32	10.63	21.27	5.38	10.77	21.53	2.31	4.62	9.25	2.34	4.68	9.36
Adult men	3.97	7.95	15.90	4.18	8.37	16.73	0.88	1.77	3.53	0.93	1.86	3.72
Children aged 5 to 12	9.46	18.92	37.84	10.09	20.17	40.34	4.11	8.23	16.45	4.38	8.77	17.54
Children aged 2 to 4	18.36	36.71	73.43	18.83	37.65	75.30	7.98	15.96	31.93	8.19	16.37	32.74

**Table 4:** Calculation of maximum safe fish consumption (in grams per day) according to the U.S. EPA (2000).

		Maximum Safe Fish Consumption (in Grams per Day) According to U.S. EPA (2000)							
		Women of Childbearing Age		Adult Men		Children Aged 5 to 12		Children Aged 2 to 4	
Common Name	Average (Hg) μg/g	Urban	Non-Urban	Urban	Non-Urban	Urban	Non-Urban	Urban	Non-Urban
Aracu	0.137	37.42	36.96	50.07	47.57	21.04	19.73	10.84	10.57
Barba Chata	2.075	2.47	2.44	3.31	3.14	1.39	1.30	0.72	0.70
Cará-açú	0.397	12.91	12.76	17.28	16.42	7.26	6.81	3.74	3.65
Coroataí	2.014	2.55	2.51	3.41	3.24	1.43	1.34	0.74	0.72
Curimatã	0.091	56.34	55.65	75.37	71.62	31.67	29.70	16.32	15.91
Dourada	0.665	7.71	7.62	10.31	9.80	4.33	4.06	2.23	2.18
Filhote/Piraíba	1.172	4.37	4.32	5.85	5.56	2.46	2.31	1.27	1.24
Jandiá	0.092	55.73	55.04	74.55	70.84	31.33	29.38	16.14	15.74
Jaraqui	0.039	131.46	129.85	175.87	167.10	73.90	69.31	38.08	37.13
Liro	0.426	12.04	11.89	16.10	15.30	6.77	6.35	3.49	3.40
Mandii	0.439	11.68	11.54	15.62	14.85	6.56	6.16	3.38	3.30
Mandubé	0.621	8.26	8.15	11.05	10.49	4.64	4.35	2.39	2.33
Matrinxã	0.13	39.44	38.95	52.76	50.13	22.17	20.79	11.42	11.14
Pacú	0.03	170.90	168.80	228.63	217.23	96.07	90.10	49.50	48.27
Pescada	0.61	8.40	8.30	11.24	10.68	4.72	4.43	2.43	2.37
Piracatinga	1.472	3.48	3.44	4.66	4.43	1.96	1.84	1.01	0.98
Pirandirá	1.062	4.83	4.77	6.46	6.14	2.71	2.55	1.40	1.36
Piranha-preta	0.401	12.79	12.63	17.10	16.25	7.19	6.74	3.70	3.61
Surubim	0.646	7.94	7.84	10.62	10.09	4.46	4.18	2.30	2.24
Tucunaré	0.706	7.26	7.17	9.72	9.23	4.08	3.83	2.10	2.05

**Table 5:** Calculation of maximum safe fish consumption (in grams per day) according to FAO/WHO (2003).

		Maximum Safe Fish Consumption (in Grams per Day) According to FAO/WHO (2003)							
		Women of Childbearing Age		Adult Men		Children Aged 5 to 12		Children aged 2 to 4	
Common Name	Average (Hg) μg/g	Urban	Non-Urban	Urban	Non-Urban	Urban	Non-Urban	Urban	Non-Urban
Aracu	0.137	86.07	85.02	225.30	214.06	48.38	45.38	24.93	24.31
Barba Chata	2.075	5.68	5.61	14.87	14.13	3.19	3.00	1.65	1.61
Cará-açú	0.397	29.70	29.34	77.75	73.87	16.70	15.66	8.60	8.39
Coroataí	2.014	5.86	5.78	15.33	14.56	3.29	3.09	1.70	1.65
Curimatã	0.091	129.58	127.99	339.18	322.27	72.84	68.32	37.53	36.60
Dourada	0.665	17.73	17.51	46.41	44.10	9.97	9.35	5.14	5.01
Filhote/Piraíba	1.172	10.06	9.94	26.34	25.02	5.66	5.30	2.91	2.84
Jandiá	0.092	128.18	126.60	335.49	318.77	72.05	67.58	37.13	36.20
Jaraqui	0.039	302.36	298.65	791.42	751.96	169.96	159.41	87.58	85.39
Liro	0.426	27.68	27.34	72.45	68.84	15.56	14.59	8.02	7.82
Mandii	0.439	26.86	26.53	70.31	66.80	15.10	14.16	7.78	7.59
Mandubé	0.621	18.99	18.76	49.70	47.22	10.67	10.01	5.50	5.36
Matrinxã	0.13	90.71	89.59	237.43	225.59	50.99	47.82	26.27	25.62
Pacú	0.03	393.07	388.24	1028.85	977.55	220.95	207.23	113.85	111.01
Pescada	0.61	19.33	19.09	50.60	48.08	10.87	10.19	5.60	5.46
Piracatinga	1.472	8.01	7.91	20.97	19.92	4.50	4.22	2.32	2.26
Pirandirá	1.062	11.10	10.97	29.06	27.61	6.24	5.85	3.22	3.14
Piranha-preta	0.401	29.41	29.05	76.97	73.13	16.53	15.50	8.52	8.31
Surubim	0.646	18.25	18.03	47.78	45.40	10.26	9.62	5.29	5.16
Tucunaré	0.706	16.70	16.50	43.72	41.54	9.39	8.81	4.84	4.72

## Data Availability

Not applicable.
